# Identification of a Compound That Disrupts Binding of Amyloid-β to the Prion Protein Using a Novel Fluorescence-based Assay*

**DOI:** 10.1074/jbc.M115.637124

**Published:** 2015-05-20

**Authors:** Emmanuel Risse, Andrew J. Nicoll, William A. Taylor, Daniel Wright, Mayank Badoni, Xiaofan Yang, Mark A. Farrow, John Collinge

**Affiliations:** From the Medical Research Council (MRC) Prion Unit and Department of Neurodegenerative Disease, University College London (UCL) Institute of Neurology, London WC1N 3BG, United Kingdom

**Keywords:** Alzheimer disease, amyloid-beta (AB), drug discovery, fluorescence anisotropy, high-throughput screening (HTS), isothermal titration calorimetry (ITC), neurodegenerative disease, prion

## Abstract

The prion protein (PrP) has been implicated both in prion diseases such as Creutzfeldt-Jakob disease, where its monomeric cellular isoform (PrP^C^) is recruited into pathogenic self-propagating polymers of misfolded protein, and in Alzheimer disease, where PrP^C^ may act as a receptor for synaptotoxic oligomeric forms of amyloid-β (Aβ). There has been considerable interest in identification of compounds that bind to PrP^C^, stabilizing its native fold and thereby acting as pharmacological chaperones to block prion propagation and pathogenesis. However, compounds binding PrP^C^ could also inhibit the binding of toxic Aβ species and may have a role in treating Alzheimer disease, a highly prevalent dementia for which there are currently no disease-modifying treatments. However, the absence of a unitary, readily measurable, physiological function of PrP makes screening for ligands challenging, and the highly heterogeneous nature of Aβ oligomer preparations makes conventional competition binding assays difficult to interpret. We have therefore developed a high-throughput screen that utilizes site-specifically fluorescently labeled protein to identify compounds that bind to PrP and inhibit both Aβ binding and prion propagation. Following a screen of 1,200 approved drugs, we identified Chicago Sky Blue 6B as the first small molecule PrP ligand capable of inhibiting Aβ binding, demonstrating the feasibility of development of drugs to block this interaction. The interaction of Chicago Sky Blue 6B was characterized by isothermal titration calorimetry, and its ability to inhibit Aβ binding and reduce prion levels was established in cell-based assays.

## Teaser

**Background:** The cellular form of the prion protein (PrP^C^) is implicated in Alzheimer disease as a receptor for amyloid-β (Aβ).

**Results:** An assay was developed capable of identifying inhibitors of Aβ binding to PrP^C^.

**Conclusion:** Chicago Sky Blue inhibits Aβ binding to PrP^C^.

**Significance:** This is the first small molecule shown to disrupt the binding of Aβ to PrP^C^.

## Introduction

Prion diseases are invariably fatal neurodegenerative diseases for which there is no effective treatment and encompass Creutzfeldt-Jakob disease in humans, as well as scrapie, bovine spongiform encephalopathy, and chronic wasting disease in animals (1). In humans, prion disease can arise sporadically, be acquired through infection, or be inherited as a result of mutations in the gene coding for the cellular form of the prion protein (PrP).^3^ Prion disease is caused by the recruitment of the normal cellular form of the protein (PrP^C^) into self-propagating polymeric forms, often referred to as PrP^Sc^, although multiple disease-related isoforms occur associated with infectivity and neurotoxicity (2–5). Although rare, prion diseases have been the subject of intense study for many years, and much of what has been learnt of their fundamental molecular processes is now being applied to other, more common neurodegenerative diseases that also involve protein misfolding and aggregation such as Parkinson and Alzheimer disease (AD) (6, 7). Recently, a more direct connection between prion protein itself and AD was suggested when PrP^C^ was identified as a receptor for amyloid-β (Aβ) oligomers, which are considered as key pathogenic species in AD (8).

Validation of PrP^C^ as a therapeutic target in prion neurodegeneration was demonstrated using conditional knock-out mouse models where depletion of neuronal PrP^C^ in adult mice with established neuroinvasive prion infection prevented neuronal loss and clinical onset, and indeed where early neuropathological and behavioral changes were reversed (9, 10). Similarly, PrP^C^ has been successfully therapeutically targeted using monoclonal antibodies, where cell cultures could be cured of prion infection (11) and treatment of prion-infected mice could prevent disease progression (12). That ligands binding to the folded domain of PrP^C^ and stabilizing its native fold would block prion propagation was supported by biophysical data (13) and a proof of principle study (14).

PrP^C^ was identified as a receptor for Aβ oligomers in a cell-based screen and shown to be required for Aβ-induced inhibition of hippocampal long-term potentiation (8). Both antibodies (8, 15–19) and knock-out mouse models (8, 17, 20, 21) have also been used to demonstrate the PrP dependence of the interaction of Aβ oligomers and its role in synaptic plasticity and memory. Although the role of PrP as a receptor for Aβ and its significance in Alzheimer disease were initially controversial (22–24), considerable data from independent laboratories have accumulated supporting the association between PrP and Aβ oligomers (25–30), whereas the earlier discrepancies could be explained by differences in the preparation of the Aβ oligomers and the models used (31). A small molecule inhibitor of the interaction of Aβ with PrP would be of obvious use in further investigation of the role of PrP in AD pathogenesis and the relative importance of other putative Aβ oligomer receptors, which now include leukocyte immunoglobulin-like receptor LilrB2 (32), EphB2 kinase (33), and immunoglobulin G Fcγ receptor II-b (FcγRIIb) (34).

Given the severe unmet clinical need in prion diseases, which are invariably fatal, there has been considerable interest in the identification of small molecules that bind to PrP^C^ and prevent its conversion to pathogenic forms as candidate therapeutics (35, 36). However, despite extensive efforts, there remain few examples of high-affinity PrP^C^ ligands, and those that have been reported generally represent poor starting points for a medicinal chemistry program to deliver a clinical candidate (14, 36). Despite the firm validation of PrP^C^ as a therapeutic target, the small molecule therapeutics for prion disease that have been demonstrated to be successful *in vivo* either target aggregated prion protein (37), a downstream target, or have an unknown molecular target (38). We therefore sought to develop an assay capable of screening large numbers of compounds to identify ones that bind to PrP^C^.

A fluorescence polarization (FP)-based assay to detect ligand binding was first established using several known biological ligands for PrP, including Aβ oligomers. Rather than attempting to directly detect small molecule-induced changes in the FP signal, we measured the ability of compounds to inhibit the binding of the Aβ oligomers. This assay was then used to screen a small collection of pharmacologically active compounds with rapid confirmation of binding using an orthologous assay for Aβ binding in an ELISA format. The binding of the only compound to show robust activity in both assays, Chicago Sky Blue 6B, was then characterized using isothermal titration calorimetry (ITC), and its activity in a cellular context was confirmed by measuring the ability of the compound to lower prion levels in a chronically prion-infected mouse neuroblastoma cell line (14).

## Experimental Procedures

Aβ_1–42_ and Aβ_1–42_ with biotin attached to Asp-1 using a 6-carbon linker (bAβ_1–42_) were synthesized and purified by Dr. James I. Elliott at Yale University (New Haven, CT). Aβ oligomers were produced as described previously (17) and contained a mixture of monomer, spherical oligomers, and protofibrils. Anti-PrP antibodies ICSM35 and ICSM18 were from D-Gen Ltd. (London, UK). The Prestwick Chemical Library compound collection of 1,200 compounds was purchased as 10 mm stock in dimethyl sulfoxide (DMSO) from Prestwick Chemical (Illkirch-Graffenstaden, France). Chicago Sky Blue 6B solid was purchased from Sigma and dissolved in DMSO 24 h prior to use.

Mutations to the wild-type human prion protein (amino acids 23–231) were generated by QuikChange mutagenesis and verified by sequencing. Prion protein with an N-terminal hexahistidine tag followed by a 23-amino acid linker and a thrombin cleavage site was expressed in *Escherichia coli* (39), solubilized in 6 m guanidine hydrochloride, and purified by affinity chromatography on a nickel-nitrilotriacetic acid column prior to undergoing stepwise oxidation and elution (40). Following elution, proteins were dialyzed for 1 h against 10 mm Tris-HCl, 100 mm sodium phosphate, pH 5.8, prior to being labeled with IANBD (*N*-((2-(iodoacetoxy)ethyl)-*N*-methyl)amino-7-nitrobenz-2-oxa-1,3-diazole) ester (Invitrogen). A 10 mm stock of IANBD ester was freshly made in dimethyl formamide and added to the protein in a 5-fold molar excess. After 1 h, the solution was filtered through a 0.45-μm filter and then dialyzed extensively against 10 mm HEPES, pH 6.5. Proteins were stored in the presence of 20% (v/v) glycerol at −80 °C and used without further purification. Purity of the proteins was confirmed by SDS-PAGE and MALDI-TOF mass spectrometry, and conformation of the proteins was confirmed by circular dichroism. Unlabeled proteins for use in ITC and ELISA experiments were purified as described previously (40), with the N-terminal hexahistidine tag being removed by proteolysis with thrombin and the proteins being purified by ion exchange chromatography prior to use.

### 

#### 

##### Circular Dichroism

Circular dichroism spectra of NBD-labeled proteins were recorded in 10 mm sodium phosphate, pH 6.5, with 2.5 μm protein in a 2-mm path length cuvette using a Jasco 715 spectropolarimeter.

##### Fluorescence Assay

NBD-labeled proteins with the N-terminal hexahistidine tag were dialyzed extensively against 10 mm HEPES, pH 6.5. After quantification using an ϵ_280 nm_ of 56,667 mol^−1^ cm^−1^, the protein was diluted in FP buffer (10 mm Hepes, 150 mm NaCl, 0.05% (w/v) Pluronic F127, pH 7.5).

To examine the binding of Aβ oligomers and anti-PrP antibodies, 45 μl of 133 nm labeled protein in FP buffer was added to 5 μl of 5 μm anti-PrP antibodies or 10 μm Aβ oligomers in a 384-well black plate (Greiner Bio-One, FLUOTRAC 200) in triplicate. The FP signal was read after 10 min using a PerkinElmer EnVision plate reader (PerkinElmer, Seer Green, UK) with excitation at 485 nm and emission at 535 nm.

15 Prestwick plates (96-well plates) each containing 80 compounds at 10 mm in 100% DMSO were diluted initially 1/10 in 100% DMSO, and then 1/10 in water. Finally, either 5 μl of compound or 5 μl of 10% (v/v) DMSO (negative control) was transferred to medium binding 384-well black plate. 40 μl of 150 nm protein was added to each well using a Multidrop Combi (Thermo Scientific). The FP signal was read after 10 min. Aβ oligomers (5 μl) were added to a final concentration of 1 μm with respect to starting Aβ monomer concentration, and the FP signal was read again after 30 min. The first reading was subtracted to the second reading, and this value was compared with the DMSO only value to identify hit compounds.

Compounds that gave >50% inhibition without a greater than 3-fold change in total intensity were retested in duplicate in the fluorescence assay in a 10-point dose-response curve starting from 100 μm, with selected compounds tested in a 15-point dose-response curve, and results were analyzed using GraphPad Prism 6 (GraphPad Software Inc.).

##### ELISA Assay

Human PrP was diluted to 1 μm in coating buffer (10 mm sodium carbonate, pH 9.6), incubated overnight in medium binding 96-well white plates (LUMITRAC 200, Greiner Bio-One), and blocked for 1 h with Superblock (Pierce, Cramlington, UK). For direct binding experiments, anti-PrP antibodies ICSM18 and ICSM35 or biotinylated Aβ oligomers were added in PBS and incubated for 15 min. After washing, dissociation-enhanced lanthanide fluorescent immunoassay (DELFIA) Eu-N1 anti-mouse antibody or DELFIA Eu-N1 streptavidin (PerkinElmer) was added and incubated for 30 min prior to the addition of DELFIA enhancement solution (PerkinElmer). For inhibition experiments, compounds were added in PBS supplemented with 1% (v/v) DMSO, 0.05% (v/v) Tween 20. After 30 min, 100 nm biotinylated Aβ oligomer was added. Plates were incubated for a further 15 min with gentle rocking prior to washing and detection with DELFIA Eu-N1 streptavidin. Following the addition of DELFIA enhancement solution, plates were incubated for 1 h at room temperature, and time-resolved fluorescence intensity was measured with an EnVision plate reader with excitation at 320 nm, and with emission being measured at 615 nm with a 160-μs delay.

##### Isothermal Titration Calorimetry

Titrations were performed in triplicate at 25 °C on a VP-ITC (MicroCal) device. After removal of the N-terminal hexahistidine tag, the proteins were extensively dialyzed into 10 mm Hepes, 150 mm NaCl, pH 6.5. In each experiment, 10 μm protein (10 mm Hepes, 150 mm NaCl, 5% (v/v) DMSO, pH 6.5) in the cell was titrated with 14 20-μl injections (at 180-s intervals) of Chicago Sky Blue at 500 μm. Data were collected and analyzed using a one-site model in the Origin software (version 7.0) provided by MicroCal.

##### Cell-based Aβ Binding Assay

Binding of Aβ oligomers to cells was measured as described previously (8), with COS-7 cells transiently transfected with pCMV6 (OriGene) expressing full-length mouse prion protein. PrP expression was confirmed by immunofluorescence. Twenty-four hours after transfection, Aβ binding was determined by co-incubation with 500 nm biotinylated Aβ oligomers with increasing concentrations of Chicago Sky Blue for 2 h at 22 °C prior to fixing. Cells were subsequently incubated in PBS for 2 h at 65 °C to inactivate endogenous alkaline phosphatase, blocked with 3% (v/v) donkey serum in PBS containing 0.1% (v/v) Triton X-100, and incubated overnight at 4 °C with NeutrAvidin-conjugated alkaline phosphatase (Thermo Scientific). Cells were washed with PBS and then with alkaline phosphatase buffer (100 mm Tris-HCl (pH 9.5), 100 mm NaCl, 5 mm MgCl_2_) to remove unbound NeutrAvidin-conjugated alkaline phosphatase, prior to staining with 5-bromo-4-chloro-3-indolyl phosphate/nitro blue tetrazolium. The number of Aβ-positive cells in each well was determined using a Bioreader® 5000-Eβ (Bio-Sys).

##### Cell-based Anti-prion Assay

Compounds were incubated for 3 days with N2a cells (PK1 subclone) (41) chronically infected with RML mouse prions at 37 °C in Opti-MEM plus penicillin/streptomycin. PrP^Sc^ levels were measured in a dot blot assay following denaturation and digestion with proteinase K, with detection using ICSM18 and visualization using a LI-COR Odyssey reader.

## Results

**FIGURE 1.**
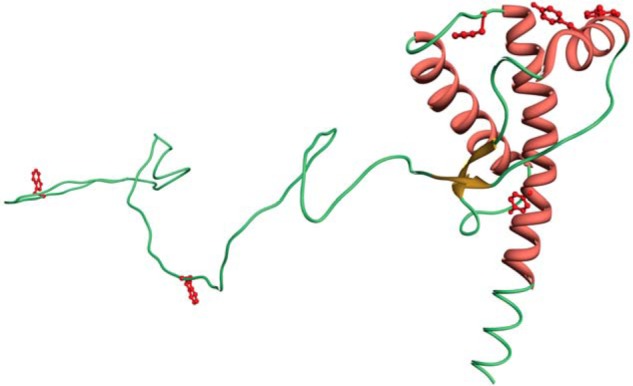
**Representation of the structure of the human prion protein showing the sites where cysteine residues were introduced to permit labeling with IANBD.**

To reduce the potential perturbation of the native protein structure caused by the introduction of a fluorescent probe, we first identified six aromatic residues that, by inspection of the available PrP structures, were surface-exposed (42), and therefore most likely to be able to accommodate the fluorophore without significantly disrupting the native structure of the protein (Fig. 1). These were then individually mutated to a cysteine residue to allow labeling with a thiol-reactive fluorescent probe. To further minimize the disruption of the protein structure, we also chose to utilize a small (*M*_r_ <400), environmentally sensitive fluorophore, namely IANBD ester.

**FIGURE 2.**
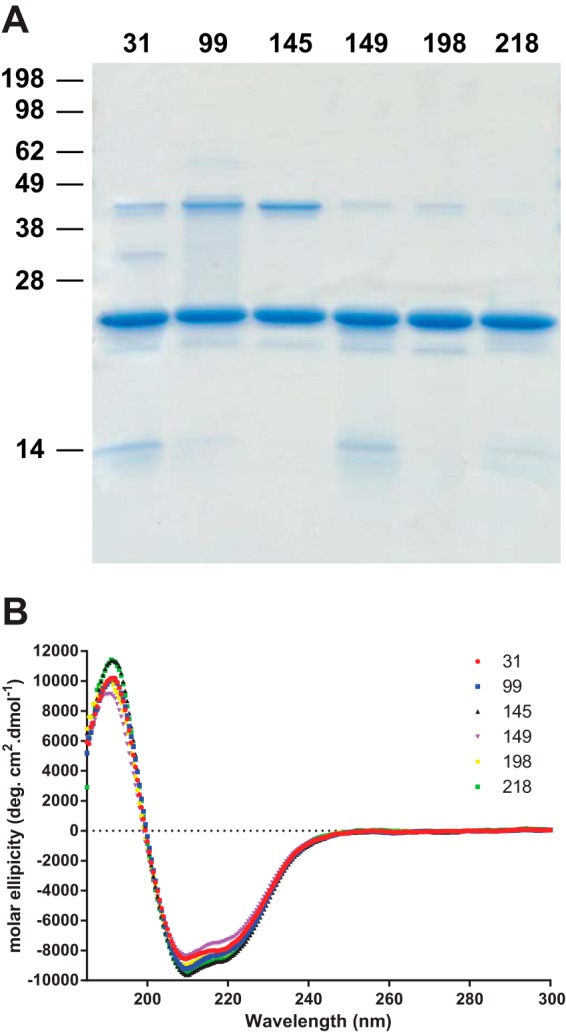
**Characterization of the NBD-labeled proteins.** *A*, Coomassie Blue-stained 16% polyacrylamide Tris-glycine gel showing NBD-labeled protein. Prior to loading, samples were heated to 95 °C for 5 min in SDS loading buffer in the absence of reducing agent. *B*, circular dichroism spectra of the PrP proteins labeled at the different sites. *deg*, degrees.

**FIGURE 3.**
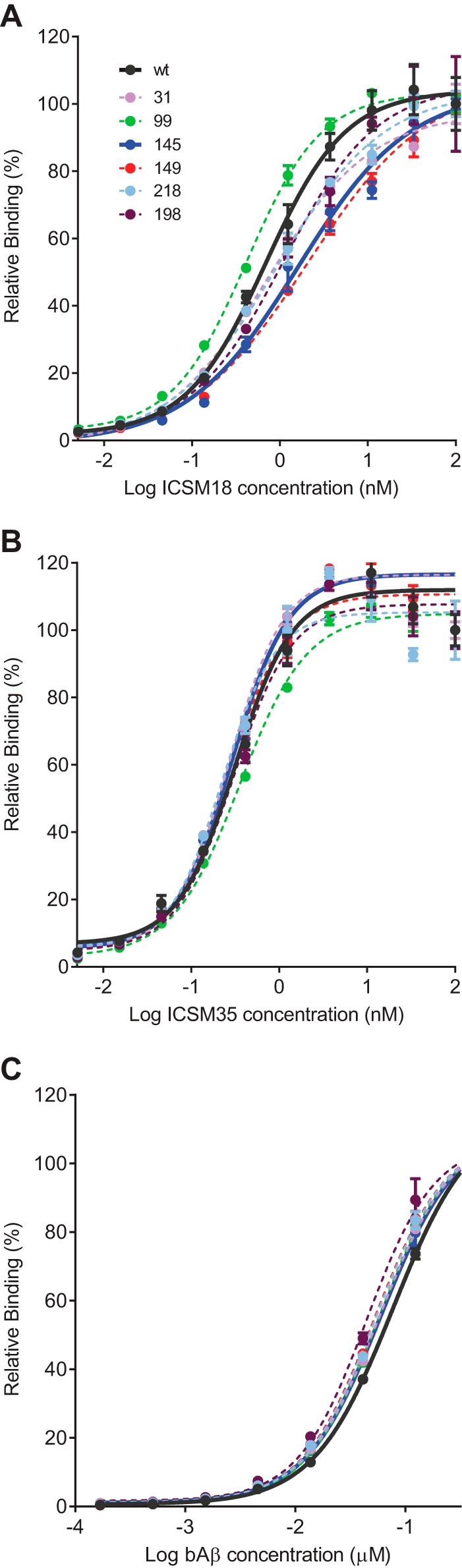
**Results for ELISA assays showing the relative binding of ICSM18 (*A*), ICSM35 (*B*), and Aβ (*C*) oligomers to the different NBD-labeled constructs.** Curves showing binding to wild-type protein and 145 construct (the construct used in the screen) are shown as *continuous lines*, and binding curves for the other proteins are shown as *dashed lines*. Assays were performed in triplicate with the mean values ± S.E. shown.

The mutant proteins were expressed in *E. coli* (39) and purified (40) as described previously prior to being labeled with IANBD ester. Despite the incorporation of an additional cysteine into a protein that already has two cysteine residues, slow oxidation of the protein encouraged formation of the most thermodynamically stable disulfide, whereas elution at a relatively low pH prevented the formation of intermolecular dimers such that following oxidative refolding on a nickel-nitrilotriacetic acid column and labeling with IANBD, > 70% of the protein was monomeric as judged by SDS-PAGE in the absence of reducing agent (Fig. 2*A*). The absence of large changes in the conformation of the protein caused by incorporation of the NBD fluorophore was confirmed by circular dichroism (Fig. 2*B*). As expected following introduction of an environmentally sensitive fluorophore, both absorbance and emission spectra of the different constructs varied considerably (data not shown). To confirm that the introduction of NBD was not perturbing the ability of the proteins to interact with biological ligands, their binding to a variety of ligands was determined: Aβ oligomers, which interact with residues 95–105 (8) and 23–31 (30), and anti-PrP antibodies ICSM18, which binds to residues 143–153 (43), located within the first α-helix of the structured domain of the protein, and ICSM35, which binds to residues 93–105 at the very N terminus of the structured domain (44) and overlapping the primary Aβ binding site (17). The affinity of the ligands was determined in an ELISA type assay, and we found less than 2-fold differences in the affinities of the mutant protein for the different ligands (Fig. 3).

Because the aim of the study was to develop an assay capable of screening large compound libraries, we decided to use FP to monitor ligand-induced changes to the fluorescence of the probe. This is because changes in the environment of the probe would be expected to result in a change to its fluorescence lifetime, thereby resulting in a change in the FP signal. This is in contrast to the majority of FP-based assays where changes to the FP signal arise from alterations to the mobility of the fluorophore normally caused by a large change in the molecular weight of the analyte. Using commonly available equipment, it was possible to read a 384-well plate in the FP assay in 1–2 min, whereas monitoring changes in the fluorescence spectra would necessitate much lengthier read times and would be more prone to compound interference.

**FIGURE 4.**
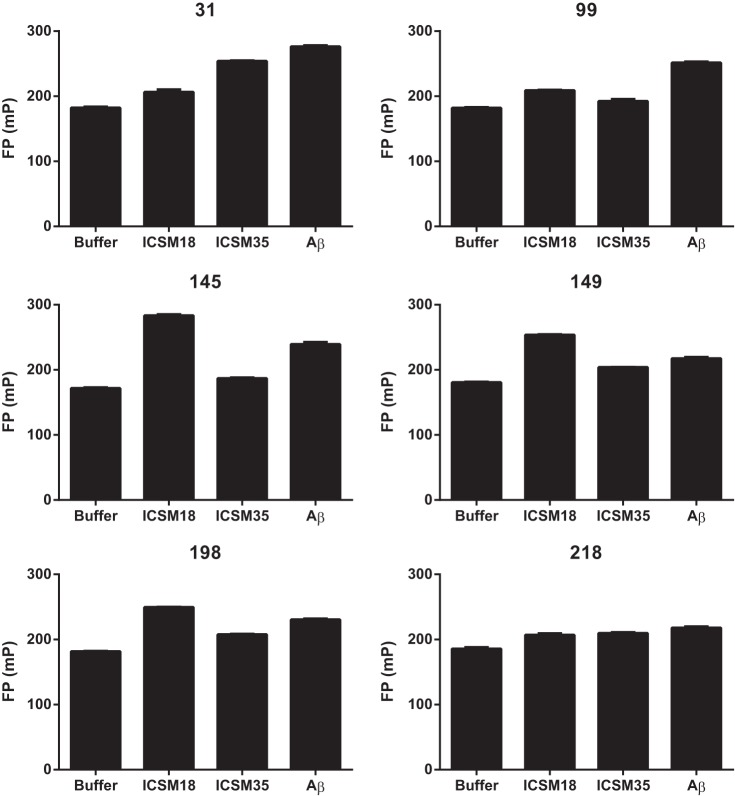
**Interaction of biological ligands with the different prion protein reporter constructs.** 120 nm labeled prion protein was incubated with 500 nm anti-prion antibodies ICSM18 and ICSM35, or with 1 μm synthetic amyloid β oligomers. Assays were performed in triplicate, with values expressed as mean ± S.E. All constructs demonstrated a statistically significant change in FP signal upon binding of all three ligands (one-way analysis of variance with Dunnett's multiple comparisons test). *mP*, millipolarization units.

To identify the optimum construct for screening for PrP ligands, we tested the ability of the proteins labeled at different sites to detect the binding of known ligands. All the constructs demonstrated a statistically significant change in FP signal upon ligand binding (one-way analysis of variance with Dunnett's multiple comparisons test), although the magnitude of these changes varied significantly (Fig. 4). Protein labeled at the N-terminal residue 31 exhibits changes in fluorescence following binding of Aβ oligomers and ICSM35, both of which bind toward the N terminus of the protein, but not after binding of ICSM18 to the structured domain. Given the proximity of Aβ and ICSM35 binding sites to residue 31, it is perhaps unsurprising that protein labeled at this position is more responsive to their binding than to ICSM18 binding. Interestingly, despite being located within the middle of the linear epitope for ICSM35, protein derivatized at position 99 showed little response upon ICSM35 binding, although did show changes in the fluorescence upon Aβ oligomer binding. This suggests that the environment of the fluorophore is little changed upon ICSM35 binding, but that binding of Aβ oligomers triggers a change in the conformation of the protein resulting in a change in the environment of the fluorophore.

Derivatization of the protein within the structured domain at positions 145,149, 198, or 218 resulted in a range of sensitivities to ligand binding. Protein labeled at position 218 was relatively insensitive to binding of any of the ligands, whereas labeling at positions 145, 149, or 198 all gave constructs that showed changes upon Aβ and ICSM18 binding, and much smaller changes with ICSM35 binding.

We selected the protein labeled at position 145 as being the most responsive to both Aβ binding and molecules that bind the structured domain (ICSM18) and then proceeded to use that protein to screen a small library of 1,200 approved drugs and pharmacological tool compounds (Prestwick Chemical Library). In the high-throughput single point screening assay, the labeled protein was incubated with compounds for 15 min, and the FP signal was measured to establish whether the compound itself was causing a change in the fluorescence of the probe. Aβ oligomers were then added, and following a further 30-min incubation, the plate was read again. The initial reading was subtracted from the reading in the presence of Aβ to give the ΔFP value, with the 24 compounds that reduced the ΔFP value by more than 50% being classified as hits. Following the initial screen, hit compounds were retested in the fluorescence assay to determine their IC_50_. Those compounds with an IC_50_ of less than 100 μm are shown in Table 1, whereas 15 of the initial hit compounds failed to show any activity in the dose-response assay.

**TABLE 1:** **IC_50_ values for hits in the fluorescence and ELISA assays**

Compound name	Fluorescence assay IC_50_ values	ELISA IC_50_ values
	μ*m*	μ*m*
Chicago Sky Blue 6B	0.41	19.7
Verteporfin	1.7	>100
Clofazimine	4.4	>100
Amphotericin B	5.9	88.8
Benserazide HCl	6.3	>100
Anthralin	8.7	>100
Propidium iodide	11.2	>100
Zafirlukast	15.4	85.7
Alfadolone acetate	31.3	>100

**FIGURE 5.**
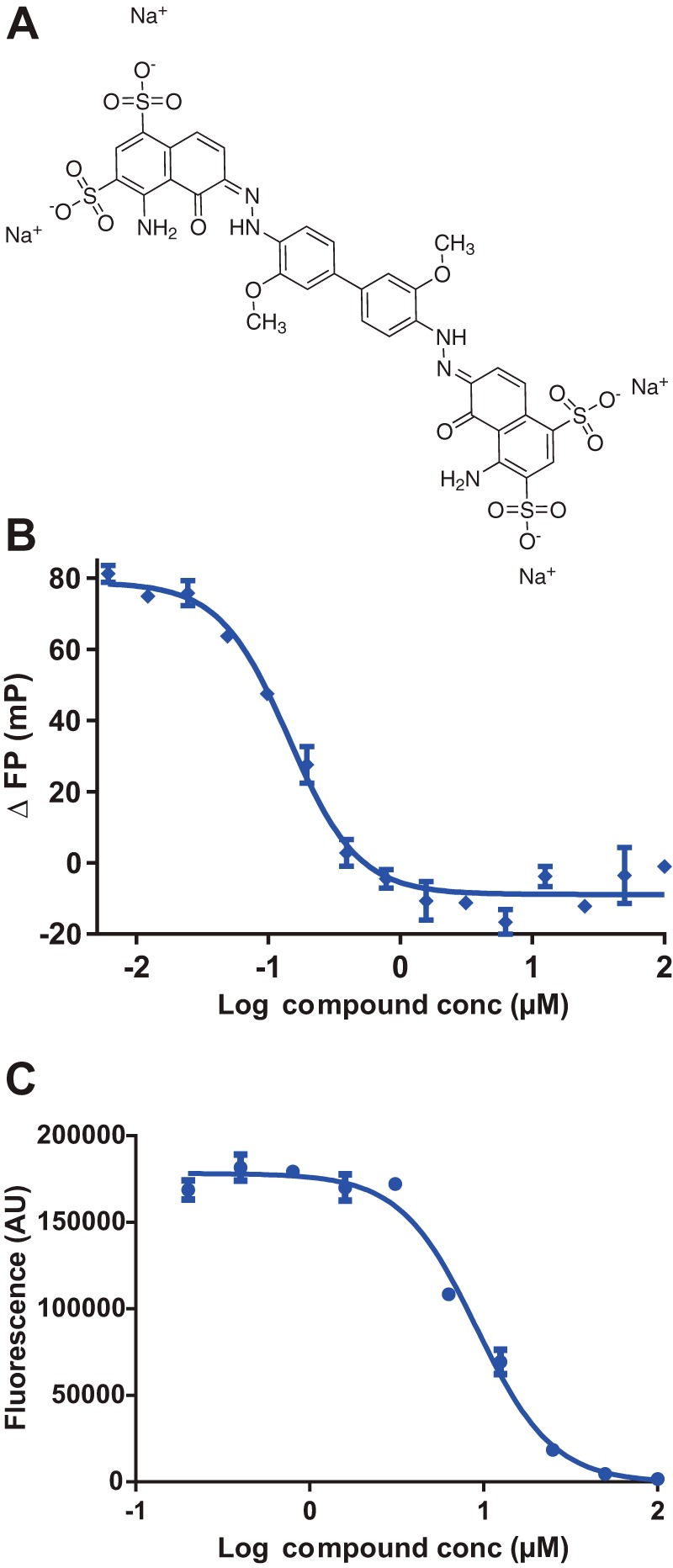
*A*, structure of Chicago Sky Blue 6B. *B* and *C*, dose-response curves for Chicago Sky Blue in fluorescence (*B*) and ELISA (*C*). Assays were performed in triplicate with the values expressed as the mean ± S.E. *mP*, millipolarization units; *conc*, concentration; *AU*, arbitrary units.

To exclude the possibility of compounds interfering in the fluorescence assay and generating false positives, the ability of the compounds to inhibit the interaction of biotinylated-Aβ oligomers with unlabeled PrP was confirmed in an ELISA assay. The only compound to show reasonable activity in both fluorescence and ELISA assays was Chicago Sky Blue (Table 1 and Fig. 5).

**FIGURE 6.**
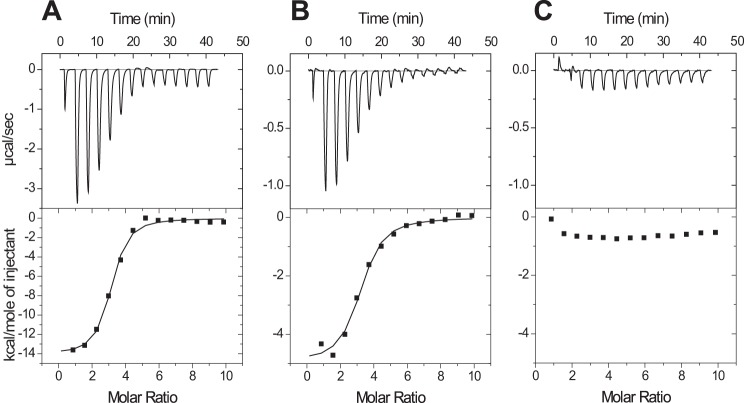
**Isothermal titration calorimetry isotherms for the interaction of Chicago Sky Blue with different length constructs of human prion protein 23–231 (*A*), 91–231 (*B*), and 119–231 (*C*).**

Because both the ELISA and the fluorescence assays involve incubating the compound in the presence of PrP and Aβ oligomers, it is possible that the compounds are binding to, and disrupting the Aβ oligomers rather than interacting with PrP. We therefore used ITC to confirm and further characterize the nature of the interaction between the compound and PrP. These experiments showed that Chicago Sky Blue binds the 23–231 construct (Fig. 6*A*) with a 3:1 ratio with a dissociation constant of 0.55 μm ± 0.04 μm (*n* = 2.80 ± 0.09, Δ*H* = −13.1 ± 0.3 kcal mol^−1^, *T*Δ*S* = −4.6 ± 0.3 kcal mol^−1^). The compound binds with similar, but slightly reduced affinity to a shorter construct containing residues 91–231 (Fig. 6*B*), which still encompasses the primary Aβ oligomer binding region of the prion protein (*K_D_* 1.43 μm ± 0.24 μm, *n* = 2.89 ± 0.18, Δ*H* = −4.8 ± 0.2 kcal mol^−1^, *T*Δ*S* = 3.2 ± 0.3 kcal mol^−1^), but shows no binding to a construct that contains only residues 119–231 (Fig. 6*C*), suggesting that the Chicago Sky Blue binding site overlaps that of the Aβ oligomers.

**FIGURE 7.**
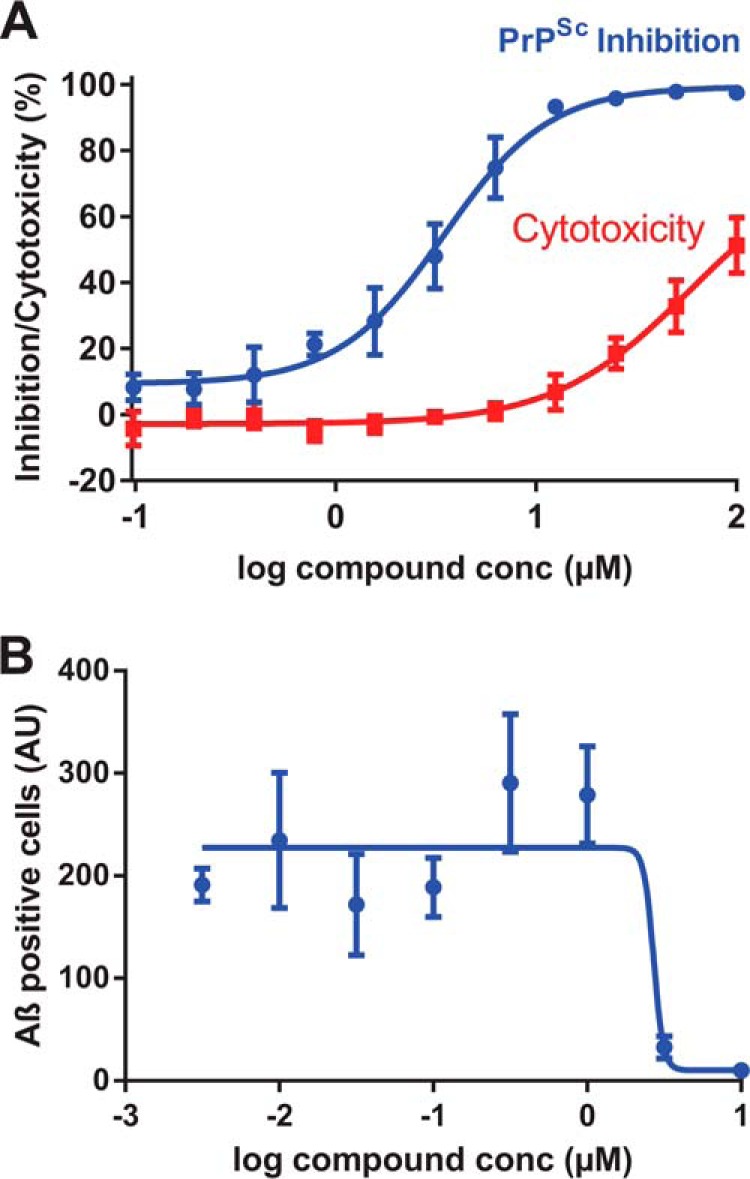
**Activity of Chicago Sky Blue in cell-based assays.** *A*, reduction in PrP^Sc^ in PK1 cells chronically infected with RML prions following 3 days of treatment with Chicago Sky Blue. *conc*, concentration. *B*, inhibition of the interaction of Aβ oligomers with COS-7 cells overexpressing mouse PrP. Data are the mean ± S.E. of four independent experiments. *AU*, arbitrary units.

To examine the ability of the Chicago Sky Blue 6B to bind to PrP when expressed in a cellular context, we used the well characterized PK1 subclone of the mouse neuroblastoma N2a cell line, which can maintain a stable long-term infection with RML prions (41). We tested the ability of Chicago Sky Blue to reduce prion levels in cells that were already chronically infected with prions. This was measured by the reduction in the levels of the disease-associated protease-resistant form of the prion protein PrP^Sc^, following incubation with the cells for 3 days. Cytotoxicity assays were performed in parallel to confirm that this reduction was not the result of a reduced cell number (14). It is clear that Chicago Sky Blue is able to reduce prion levels in cells with an IC_50_ similar to that observed in the FP assay, without causing significant cytotoxicity (Fig. 7*A*). Finally, we confirmed Chicago Sky Blue's ability to inhibit Aβ oligomer binding to cells overexpressing PrP using transiently transfected COS-7 cells (Fig. 7*B*), the system originally used to identify the interaction between PrP and Aβ oligomers (8).

## Discussion

Neurodegenerative diseases such as Alzheimer disease present a huge and increasing burden on healthcare systems worldwide. Despite the increasing prevalence associated with an aging society, there remains no effective disease-modifying treatment (45). Similarly, although the numbers affected by prion disease are far smaller, there is also no effective treatment for these invariably fatal neurodegenerative diseases. AD, rather than being a unitary disease entity, is better considered as a clinicopathological syndrome in which multiple genetic and environmental factors are involved in both its onset and its clinical progression. However, amyloid-β protein aggregation is nevertheless considered to be central, and water-soluble, non-fibrillar, Aβ assemblies are closely linked to memory impairment (46). The development of disease-modifying treatments for AD remains a challenging but hugely important goal exemplified by the recent high-profile failures of a number of clinical trials to meet their primary end point (47). Novel therapeutic strategies are clearly needed, and the identification of PrP as a receptor for toxic Aβ oligomers provides a tractable therapeutic approach, and indeed one in which the intense recent focus on targeting PrP in prion disease has established a firm experimental foundation (35).

Here we report the development of a high-throughput screen capable of identifying drug-like molecules that bind to PrP and inhibit binding of Aβ oligomers. A significant proportion of the initial hits (15/24) failed to repeat in the dose-response assay. This surprisingly high number may reflect the difficulties inherent in compound and liquid handling for single point assays within an academic environment, especially when the assays require multiple additions of reagents. Of the nine compounds that showed activity in the dose-response fluorescence assay, six showed no inhibition of Aβ binding in the ELISA assay at concentration up to 100 μm. This may reflect the more stringent nature of the assay and the irreversible nature of the binding of Aβ oligomers to high density PrP when immobilized on a solid surface (30), a suggestion supported by the significantly higher IC_50_ values seen for those compounds that did show activity in the ELISA assay. There is also a good correlation for the affinity of Chicago Sky Blue for PrP measured in solution as determined by the fluorescence assay (IC_50_ 0.41 μm) when compared with a *K_D_* of 0.55 μm, as determined by ITC. The utility of the fluorescence assay to screen large numbers of compounds followed by the ELISA assay to confirm the interaction was demonstrated by the observation that the remaining hit compound, Chicago Sky Blue, interacted not only with recombinant PrP, but also with PrP in a cellular context: being able to both reduce prion levels in chronically infected cells without causing cytotoxicity and inhibit the binding of Aβ oligomers to cells overexpressing PrP. Chicago Sky Blue has previously been shown to inhibit the DNA recombinase Rad51 (48) and glutamate uptake into synaptic vesicles (49), and is clearly not suitable for the treatment of either prion or Alzheimer disease or a promising starting point for a medicinal chemistry program. It does however represent a useful tool compound for further dissecting the interaction of Aβ oligomers with PrP in both cellular and *ex vivo* models. The fluorescence assay described is suitable for high-throughput screening of large chemical libraries, and in conjunction with the ELISA assay to rapidly identify false positives, could enable the identification of more promising lead compounds. The use of small, conformationally sensitive fluorescent probes to screen for ligand binding may also find wider application especially in the field of inhibiting protein-protein interactions where there are no readily accessible enzymatic activities to assay.

It is interesting to note that although ICSM35 and Aβ oligomers bind to a similar region of the protein, they appear to stimulate different changes to the conformation of the protein, as judged by the different changes to the FP signal. This suggests that it is unlikely that targeting PrP therapeutically with antibodies will elicit the same, neurotoxic response as Aβ oligomer binding, a finding supported by recent *in vivo* data (17, 19, 50).

## Footnotes

3 The abbreviations used are:

PrP: prion protein
PrP^C^: cellular form of the prion protein
PrP^Sc^: pathogenic (scrapie) form of the prion protein
Aβ: amyloid-β
AD: Alzheimer disease
IANBD: (*N*-((2-(iodoacetoxy)ethyl)-*N*-methyl)amino-7-nitrobenz-2-oxa-1,3-diazole) ester
NBD: 12-(*N*-methyl-*N*-(7-nitrobenz-2-oxa-1,3-diazol-4-yl))
ITC: isothermal titration calorimetry
DMSO: dimethyl sulfoxide
DELFIA: dissociation-enhanced lanthanide fluorescent immunoassay
FP: fluorescence polarization.
